# Investigation on Reaction Sequence and Group Site of Citric Acid with Cellulose Characterized by FTIR in Combination with Two-Dimensional Correlation Spectroscopy

**DOI:** 10.3390/polym11122071

**Published:** 2019-12-12

**Authors:** Zijing Cai, Bolin Ji, Kelu Yan, Quan Zhu

**Affiliations:** 1 College of Chemistry, Chemical Engineering and Biotechnology, Donghua University, Shanghai 201620, China; 2180633@mail.dhu.edu.cn (Z.C.); klyan@dhu.edu.cn (K.Y.); qzhu@dhu.edu.cn (Q.Z.); 2 National Engineering Research Center for Dyeing and Finishing of Textiles, Donghua University, Shanghai 201620, China

**Keywords:** anti-wrinkle, mechanism, citric acid, yellowing, two-dimensional correlation spectroscopy

## Abstract

Cotton fabrics are prone to wrinkles and can be treated with citric acid (CA) to obtain good anti-wrinkle properties. However, the yellowing of the CA-treated fabrics is one big obstacle to the practical application of citric acid. The changing sequence order of CA anhydride and unsaturated acid (the reason for yellowing), such as aconitic acid (AA), has not been investigated. Herein, Fourier transform infrared (FTIR) spectroscopy, two-dimensional correlation spectroscopy (2Dcos), and Gaussian calculation were employed to characterize the reaction mechanism between CA with cellulose. FTIR spectra of the CA-treated fabrics heated under different temperatures were collected and further analyzed with 2Dcos. The results indicated the changing sequence order: 1656 cm^−1^→1784 cm^−1^→1701 cm^−1^, (“→” means earlier than), i.e., unsaturated acid→anhydride→ester. Moreover, a change of Gibbs free energy (ΔG) showed that trans-AA (ΔG = −22.10 kJ/mol) is more thermodynamically favorable to be formed than CA anhydride 1 (ΔG = −0.90 kJ/mol), which was proved by Gaussian computational modeling. By taking cellobiose as a model of cellulose, the ΔG results proved that O(6)–H(6) on the glucose ring is the most likely hydroxyl to react with anhydride originated from CA or AA, especially with the terminal carbonyl group.

## 1. Introduction

Cellulose is one of the most abundant renewable natural fibers on the earth [1] and has been widely used for apparels. The cellulose-based derivatives have also played an important role in various fields, including food packaging [2,3] and adsorbent materials [4,5]. Usually, cotton fabrics are chemically modified before practical use to obtain specific functions, such as flame-retardant [6], anti-bacterial [7,8], hydrophobic [9] and anti-wrinkle properties [10,11], and so on. The easy wrinkling of cotton fabrics due to the cellulose chain movement brings a great obstacle to their applications [12]; therefore, anti-wrinkle finishing has always attracted the interests of researchers, usually forming covalent crosslinkage between different cellulose chains by chemical agents. However, the dominant agents are still the formaldehyde-based compounds at present (especially the dimethylol dihydroxyethyleneurea (DMDHEU)), which will release carcinogenic formaldehyde during manufacturing, storage, and wearing [13,14]. Alternatively, formaldehyde-free agents have been extensively investigated with the hope of replacing formaldehyde-based compounds, and polycarboxylic acids (PCAs) are regarded as the most promising ones. Among PCAs, 1,2,3,4-butanetetracarboxylic acid (BTCA) [11,15,16], citric acid (CA) [17,18,19] and 3,3′,4,4′-benzophenonetetracarboxylic acid (BPTCA) [20,21] show outstanding performance. Furthermore, CA is a cheap and renewable compound, but it will result in yellowing of the treated fabrics [17,18].

Yang confirmed the formation of various unsaturated acids from CA in the anti-wrinkle finishing process [18] and adopted polyols to improve the whiteness index of the CA-treated fabrics [17]. Consequently, the whiteness index of the CA-treated fabrics was improved by polyols, especially xylitol, due to the dicitrate that is formed between CA and polyols being more thermally stable than CA. However, this method means more chemicals being required for the anti-wrinkle finishing of cotton fabrics and brings a greater burden on the environment.

Hydrogen peroxide (H_2_O_2_) is a strong and green oxidant that can break the C=C bonds, which is the basic principle for the bleaching of raw cotton fabrics. Similarly, H_2_O_2_ was employed to bleach the CA-treated fabrics under alkaline conditions (alkaline post-bleaching) [22] or ultraviolet B conditions (UVB post-bleaching) [23], which are aimed at improving the whiteness index. Interestingly, the whiteness index of the CA-treated fabrics was improved, but the anti-wrinkle properties decreased a little. Luo [24] reported that the active agent N-[4-(triethylammoniomethyl)benzoyl] caprolactam chloride (TBCC) could be used in H_2_O_2_ bleaching of the CA-treated fabrics at a lower temperature and near-neutral conditions (TBCC post-bleaching). Although TBCC post-bleaching improved the whiteness index, the strength retention of the bleached fabrics decreased. The application of TBCC is also restricted because of its poor solubility and high price.

It has been accepted that yellowing is attributed to the formation of unsaturated acids, and CA should form the active intermediates of anhydrides before reacting with cellulose, both of which are resulted from the dehydration of CA at an elevated temperature [17,19]. However, the issues related to whether unsaturated acids are formed earlier or later than anhydrides and which sites of CA carboxyl and cellulose hydroxyl participate in the reactions have not been clarified. Therefore, it is important to clarify the changing sequence order of anhydride and unsaturated acid in order to fully understand the reaction mechanism and further develop more effective strategies to solve the yellowing problem. The clarification of reaction sites will provide a direction to develop new anti-wrinkle finishing agents with designed structures.

2Dcos was firstly proposed by Noda [25] as a powerful mathematical method to analyze the complex molecular spectroscopy. With the assistance of 2Dcos technology, some overlapped peaks in the original FTIR spectra can be well separated. Moreover, FTIR-2Dcos can be used to determine the response order of functional groups in reactions [26]. 2Dcos has been widely applied in the analyses of hydrogen bonds (H-bonds) regarding changes of cellulose and cellulose diacetate [27,28] as well as the phase transition of materials [29].

In this study, FTIR spectra of the reactions between CA and cellulose heated under consecutive increasing temperatures were obtained and further analyzed with 2Dcos. The changing sequence order of formation of anhydride and unsaturated acid from the dehydration of CA was carefully investigated. The reaction sites including carboxyls on CA and hydroxyls on cellobiose molecules were analyzed from the viewpoint of reaction thermodynamics by Gaussian computational modeling.

## 2. Materials and Methods

### 2.1. Materials

Plain woven pure cotton fabrics (14.6 tex × 14.6 tex, 117 g/m^2^), which were desized, scoured, bleached, and mercerized in advance, were provided by Hualun Printing & Dyeing Co., Ltd. (Shanghai, China). Citric acid (CA) and sodium hypophosphite (SHP) were both analytical agents that were purchased from Sinopharm Chemical Reagent Co., Ltd. (Shanghai, China). Potassium bromide (KBr) was spectral grade agent and purchased from Tianjin Botianshengda Technology Development Co., Ltd. (Tianjin, China).

### 2.2. Fabric Treatment

The fabrics were dipped into a finishing bath twice containing CA (7.0% wt) and SHP (3.9% wt) and then nipped by a padder (Xiamen Rapid Precion Machinery Co., Ltd., Xiamen, China) to obtain a wet pickup about 95%. After being dried at 80 °C for 5 min, the predried fabrics were cured at different temperatures for 3 min, washed in tap water for 3 min to remove the unreacted CA and SHP, and dried at 80 °C for 5 min again before tests of the anti-wrinkle properties. Alternatively, the predried fabrics were cut into fine powders for further use.

### 2.3. FTIR and 2Dcos

The powders of the predried fabrics (2.0 mg) were mixed with 200.0 mg of dried KBr and then pressed to be a transparent tablet. The tablet was heated in a solid transmission accessory (Pike Technologies Inc., Madison, WI, USA) set up into the instrument (Nicolet iS10, Thermo Fisher Scientific, Waltham, MA, USA) from 40–210 °C at a rate of 2 °C/min, and the FTIR spectra of samples were collected every other 2 °C.

2Dcos analyses of the FTIR spectra were operated by 2D Shige v.1.3 software (Shigeaki Morita, Kwansei-Gakuin University, Nishinomiya, Japan, 2004−2005). The final contour maps were obtained by an Origin Program v.8.0, and the pink colors and grey colors indicated positive and negative intensities, respectively.

### 2.4. Anti-Wrinkle Properties

Before measurement, fabrics were stored for at least 4 h in a condition room with temperature (21 ± 1) °C and relative humidity (65 ± 2)%, respectively. The wrinkle recovery angle (WRA) of fabrics was measured with a SDL Atlas crease recovery tester (SDL Atlas Ltd., Rock Hill, SC, USA) according to the American Association of Textile Chemists and Colorists (AATCC) Testing Method 66-2008. Tear strength was tested with a YG(B) 033D digital tearing instrument (Darong Instrument Co., Ltd., Wenzhou, China) according to the American Society of Testing Materials (ASTM) method D1424-2009. The whiteness index of fabrics was obtained by a Datacolor 650 instrument (Datacolor Inc., Lawrenceville, NJ, USA) according to the AATCC Testing Method 11-2005.

### 2.5. Gaussian Calculation

**Scheme 1:**
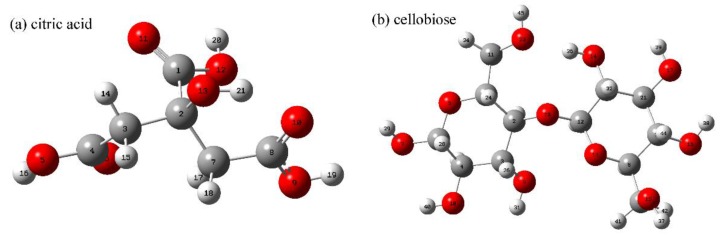

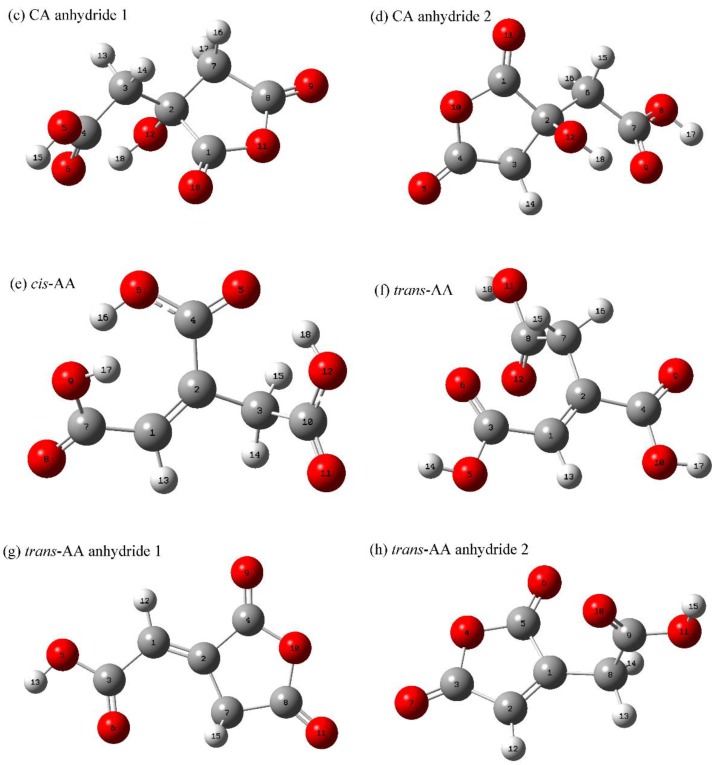
Chemical structures of different compounds.

The structures of compounds (Scheme 1) were built with a ChemBioOffice Ultra 2010 software (Perkin Elmer, Inc., Waltham, MA, USA) and were processed with MM2/Minimize energy program in sequence. The geometry of a compound was optimized with a Gaussian 09W software (Gaussian, Inc., Wallingford, CT, USA) in the density functional theory (DFT) unrestricted B3LYP/6-31G(d) level, and furthermore in the DFT unrestricted B3LYP/6-31G(d,p) level [30,31,32]. The frequency calculation confirmed no imaginary frequency. The calculated results and the structures of compounds were obtained with GaussView 5.0 software (Gaussian, Inc., Wallingford, CT, USA).

## 3. Results

### 3.1. Effects of Temperature on Peak Intensities

**Figure 1:**
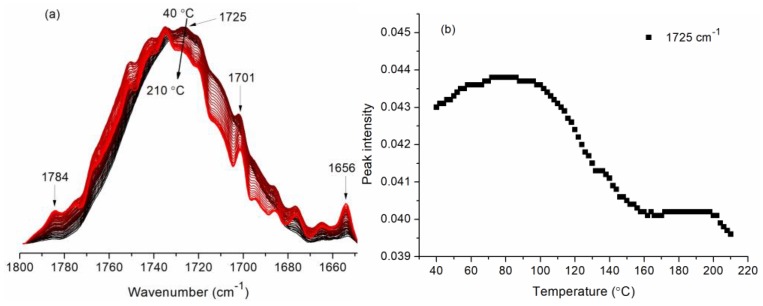
(**a**) Fourier transform infrared (FTIR) spectra and (**b**) peak intensity change of the CA treated fabrics in the range of 40–210 °C.

The Fourier transform infrared (FTIR) spectra of fabrics heated under different temperatures were collected, and the peak intensity at 1725 cm^−1^, which was attributed to the absorbance of carboxyl carbonyl (C=O) of CA on the fabrics, was statistically analyzed. Figure 1a indicates that the peak at 1725 cm^−1^ shifts to 1727 cm^−1^ with the temperature rising due to the cleavage of H-bonds, and that the peak intensity related to the carbonyl absorbance changes with the rising temperature. The peak intensity at 1725 cm^−1^ firstly increases due to the highest value and levels off at about 74–84 °C (Figure 1b), which may be attributed to the removal of the water molecules absorbed by the cellulose. The peak intensity decreases with temperature arising to 160 °C and then levels off, which is due to the reactions between CA and cellulose. Above 200 °C, the peak intensity decreases further. Considering that the predried fabrics were heated at 80 °C (Section 2.2), the FTIR spectra between 80 °C and 210 °C were selected for the following analysis.

**Figure 2:**
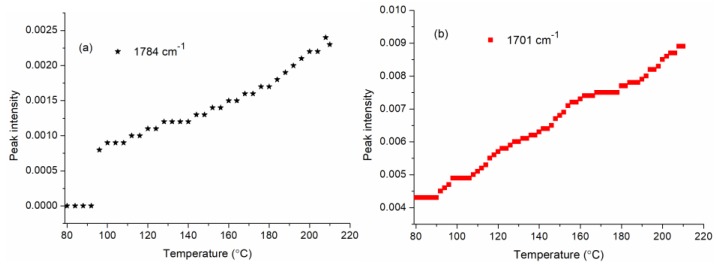
Anhydride (**a**) and carboxyl (**b**) intensities of the citric acid (CA)-treated cotton fabrics heated under 80–210 °C.

Figure 2 shows that the peak intensities at 1784 cm^−1^ and 1701 cm^−1^, which are attributed to absorbance of anhydride and ester bond, respectively, increase overall with temperature rising. This increase is because the higher temperature is beneficial to the formation of CA anhydride and the esterification between anhydride and cellulose [19]. It was noticed that the anhydride intensity is lower than the ester bond intensity, and the possible reason is that active anhydride reacts with cellulose quickly at heated conditions [19].

### 3.2. Reaction Mechanism

CA should form active anhydride intermediates before reacting with cellulose [17,19]. However, the hydroxyl in CA molecule can also dehydrate with the adjacent α-hydrogen (α-H) under a high temperature to form unsaturated acid, which is the reason for the yellowing of CA-treated fabrics. Therefore, it is important to clarify the changing sequence order of anhydride and unsaturated acid in order to fully understand the reaction mechanism and further develop more effective strategies to solve the yellowing problem. Firstly, the absorbance peaks were tentatively assigned (Table 1).

**Figure 3:**
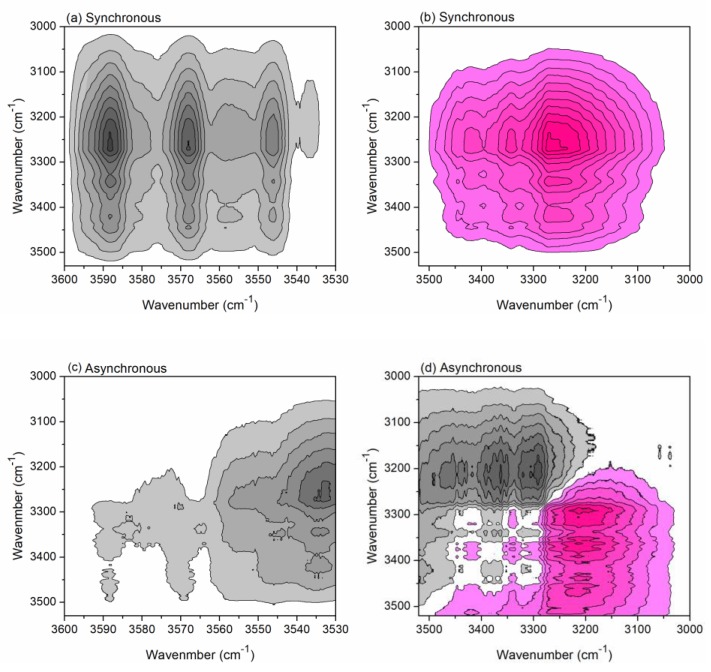
Two-dimensional correlation spectroscopy (2Dcos) synchronous (**a**,**b**) and asynchronous (**c**,**d**) maps of cotton fabrics treated with CA heated under 80–210 °C in the range of 3600–3000 cm^−1^.

Overall, there are mainly three sites of hydroxyls on a glucose ring of cellulose, i.e., O(2)–H(2) (the 2-hydroxyl group), O(3)–H(3) (the 3-hydroxyl group), and O(6)–H(6) (the 6-hydroxyl group). With the assistance of two-dimensional correlation spectroscopy (2Dcos), the changing sequence order of different absorbance peaks of hydroxyls can be identified. According to Noda’s rule [25], if the cross peak (ν1, ν2) (wavenumber ν1 > ν2) shows the same signs in the synchronous and asynchronous contour maps (both positive or negative), the change of ν1 is earlier than that of ν2; if there are different signs, the change of ν2 is earlier. For example, the cross peak of (3544 cm^−1^, 3446 cm^−1^) shows negative signs in both the synchronous map (Figure 3a) and the asynchronous map (Figure 3c); thus, the change of 3544 cm^−1^ is earlier than that of 3446 cm^−1^. Through careful analyses of the cross-peak signs in Figure 3, it can be concluded that 3544 cm^−1^→3271 cm^−1^→3334 cm^−1^→3417 cm^−1^→3446 cm^−1^ (“→” means earlier than, because the peaks shift with temperature rising, the wavenumbers were obtained from the original FTIR spectrum at 80 °C), i.e., ν(O–H) (free)→ν(O–H) (weak hydrogen bond) → ν(O(3)–H(3)…O(5)) (intrachain)→ν(O(6)–H(6)…O(3)) (interchain)→ν(O(2)–H(2)…O(6)) (intrachain) (Table 1). This can be explained by the fact that the water molecules absorbed by cellulose will be removed with the temperature rising, and free hydroxyls were released. Afterwards, the H-bonds between hydroxyls were broken up in sequence [27].

**Figure 4:**
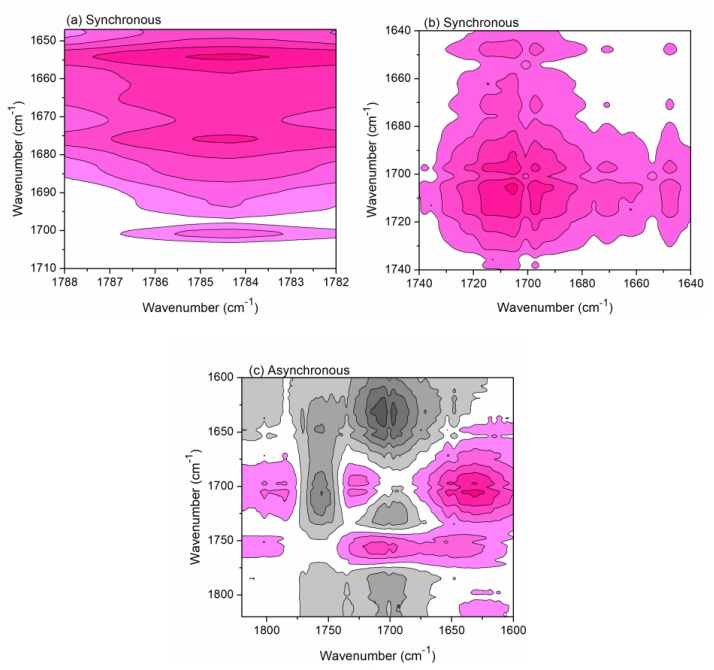
2Dcos synchronous (**a**,**b**) and asynchronous (**c**) maps of cotton fabrics treated with CA heated under 80–210 °C in the range of 1820–1600 cm^−1^.

In the following, the changing sequence order of formation of anhydride and unsaturated acid from CA was investigated. According to Noda’s rule [25], it can be concluded from Figure 4 that the changes of peaks is as follows: 1656 cm^−1^→1784 cm^−1^→1701 cm^−1^, i.e., ν(C=C) (unsaturated polycarboxylic acid)→ν(C=O) (anhydride)→ν(C=O) (ester). Therefore, the formation of unsaturated acid is earlier than that of anhydride, and then the formed anhydride esterifies with cellulose hydroxyls.

### 3.3. Theoretical Calculations

The reaction site in a molecule is related to its frontier molecular orbitals [36]. A narrower energy barrier (ΔE) between the highest occupied molecular orbital (HOMO) of molecule A and the lowest unoccupied molecular orbital (LUMO) of molecule B is beneficial to the reactivity. Gaussian calculations were conducted, and cellobiose was selected as a model molecule of cellulose for calculations.

When cellobiose reacts with acid or acid anhydride, the HOMO of cellobiose should attack the LUMO of acid or acid anhydride. The ΔE values (Table 2) show that the reactivity between cellobiose with CA anhydride is more active than with CA. Similarly, the reactivity between cellobiose with trans-AA anhydride 1 (or 2) is more active than with trans-AA, but less active than with cis-AA. However, the formation of cis-AA from CA is thermodynamically forbidden, and detailed information will be discussed later. This confirms that for PCAs, the anhydride should be firstly formed before reacting with cellulose, which provides another proof of the results (changing sequence order: 1784 cm^−1^→1701 cm^−1^) in Section 3.2. Compared with CA anhydride, the reactivity of trans-AA anhydride 1 (or 2) is more active to react with cellobiose. Trans-AA anhydride 1 is a little more active than trans-AA anhydride 2. It should be noticed that the locations of HOMO and LUMO indicate that CA can form both CA anhydride and aconitic acid, and that all three kinds of hydroxyls contribute to the HOMO (Table 2).

**Figure 5:**
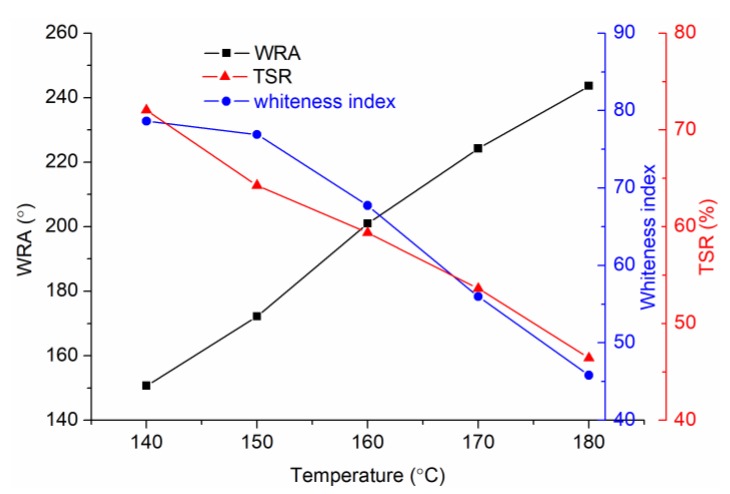
Anti-wrinkle properties of the CA-treated fabrics cured under different temperatures.

**Scheme 2:**
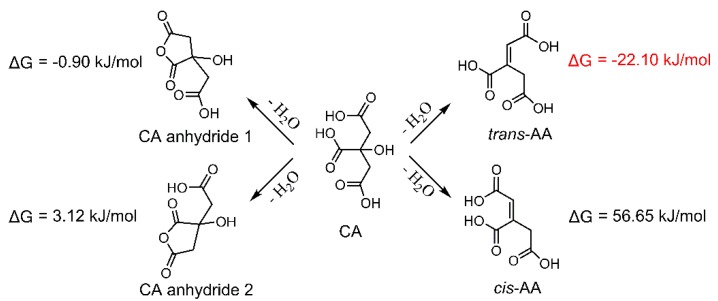
Possible products of the dehydration of CA at 170 °C.

**Scheme 3:**
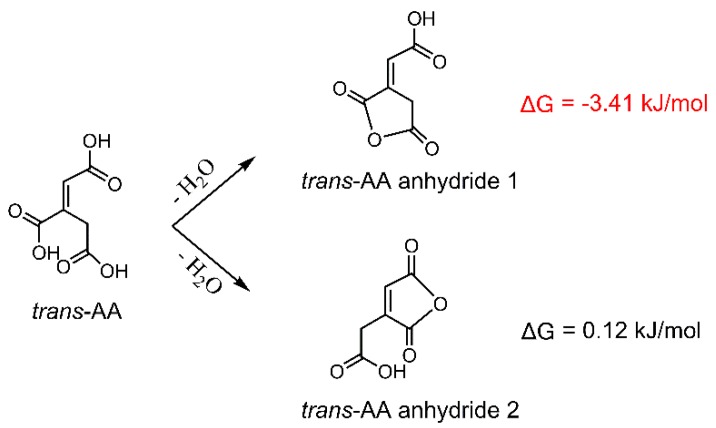
Possible products of the dehydration of trans-AA.

The anti-wrinkle properties of CA-treated fabrics are shown in Figure 5. When the fabric is cured at 170 °C, the wrinkle recovery angle (WRA) reaches a relatively high value, and the tear strength retention (TSR) is still as high as 56%. The whiteness index always decreases with the temperature rising. Therefore, 170 °C was selected as the optimal curing temperature and for the following calculation in the thermodynamic analyses. Under the 170 °C heating, CA is more thermodynamically favorable from the viewpoint of change of Gibbs free energy (ΔG) to form trans-AA (more negative ΔG) (Scheme 2), which may explain the earlier change of the C=C bond, as discussed in Figure 4. To the contrary, cis-AA is very difficult to be formed at the tested conditions, and this is why the reaction between cis-AA and cellulose is not favorable, as discussed in Table 2. Trans-AA can also form anhydride by dehydration. Scheme 3 shows that trans-AA is more prone to form anhydride 1 (more negative ΔG) compared with anhydride 2, i.e., the C=C bond is not involved in the anhydride ring. By comparing Scheme 2 and Scheme 3, trans-AA anhydride 1 (ΔG = −3.41 kJ/mol) is easier to be formed than CA anhydride 1 (ΔG = −0.90 kJ/mol). All in all, when CA is used for the anti-wrinkle finishing of cotton fabrics, the possible reaction process is that trans-AA is firstly formed as a result of CA dehydration, and then trans-AA anhydride 1 is formed; the formation of CA anhydride is later than that of trans-AA. In other words, for the CA-treated fabrics, yellowing is earlier than anhydride formation.

**Scheme 4:**
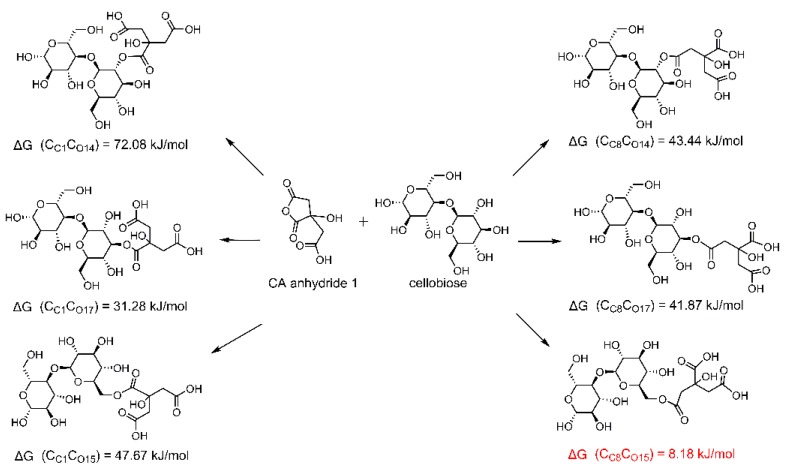
Possible reactions between CA anhydride 1 and cellobiose.

**Scheme 5:**
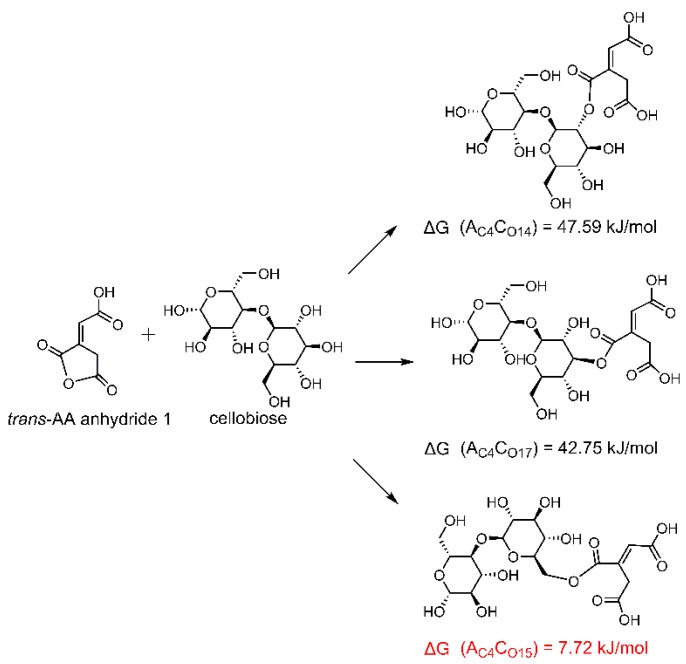
Possible reactions between trans-AA anhydride 1 and cellobiose.

Furthermore, the reaction sites of carboxyl on CA molecules and hydroxyl on cellobiose were investigated in detail. The ΔG values of possible reactions between cellobiose and CA anhydride 1 or trans-AA anhydride 1 were obtained. It can be seen in Scheme 4 that C8 on CA anhydride 1 is more prone to react with O15 on cellobiose (i.e., O(6)–H(6)) (a smaller ΔG). After that, the other two carboxyls on CA molecule can still form a 5-member cyclic anhydride by dehydration to esterify with one hydroxyl further; thus, a crosslinkage can be built up between different cellulose chains by ester bonds and eventually improve the anti-wrinkle properties of the treated cotton fabrics. If C8 reacts with cellulose, O(2)–H(2) (ΔG = 43.44 kJ/mol) and O(3)–H(3) (ΔG = 41.87 kJ/mol) show similar reactivity. For trans-AA anhydride 1, C4 is more prone to react with O15 on cellobiose (Scheme 5), and O(2)–H(2) and O(3)–H(3) show similar reactivity. After reaction, the other two carboxyls can theoretically form a six-member cyclic anhydride to react with one hydroxyl. Both Scheme 4 and Scheme 5 prove that the O(6)–H(6) on cellulose is the most possible one to react with anhydrides, and trans-AA anhydride 1 may also benefit to improve the anti-wrinkle properties of CA-treated fabrics. Since the ΔG between cellobiose with trans-AA anhydride 1 (Scheme 5) is lower than with CA anhydride 1 (Scheme 4) (indicating that trans-AA anhydride 1 is more thermodynamically favorable to crosslink with cellulose than CA anhydride 1, and consequently the yellowing substance trans-AA was fixed onto cellulose), it is suggested that the yellowing of CA-treated fabrics can be resolved after the completion of anti-wrinkle finishing, which is consistent with the results obtained from 2Dcos analyses (reaction sequence order: trans-AA→anhydride→ester).

C_C8_C_O15_ means the connection between the number 8 carbon atom on CA anhydride 1 with the number 15 oxygen atom on cellobiose. Others show the similar meanings. The atomic number can refer to Scheme 1.

## 4. Conclusions

In this study: FTIR combined with 2Dcos was employed to characterize the changing sequence order of anhydride and unsaturated acid, which were both generated from the dehydration of CA during the process of anti-wrinkle finishing of cotton fabrics. It confirmed the changing sequence order with temperature rising: ν(O(3)–H(3)…O(5)) (intrachain)→ν(O(6)–H(6)…O(3)) (interchain)→ν(O(2)–H(2)…O(6)) (intrachain). The formation of unsaturated trans-AA is earlier than that of CA anhydride, which is earlier than esterification (i.e., 1656 cm^−1^→1784 cm^−1^→1701 cm^−1^). This was also proved with thermodynamic computational modeling. In addition, the trans-AA anhydride 1 (ΔG = −3.41 kJ/mol) was more prone to be formed compared with the trans-AA anhydride 2 (ΔG = 0.12 kJ/mol). As for the hydroxyls on cellulose, O(6)–H(6) was the most likely one to firstly esterify with CA anhydride 1 or trans-AA anhydride 1. Moreover, the terminal carboxyl on the chemical structure of acid molecule would be the most likely reaction site in the first esterification with anhydride. Considering the higher reactivity of trans-AA anhydride 1 than CA anhydride 1 by showing a smaller ΔG for the reaction regarding not only the formation but also the esterification with cellulose, it was proposed to solve the yellowing problem after the anti-wrinkle finishing of cotton fabrics. This study is expected to motivate further understanding of the reaction mechanism between CA with cellulose and the development of effective strategies to solve the yellowing problem of CA-treated cotton fabrics.

## Figures and Tables

**Table 1:** Assignments of different absorbance peaks.

Wavenumber (cm^−1^)	Tentative Assignments [27,33,34,35]
3544	*ν*(O–H) (free)
3446	*ν*(O(2)–H(2)…O(6))(intrachain)
3417	*ν*(O(6)–H(6)…O(3)’) (interchain)
3334	*ν*(O(3)–H(3)…O(5)) (intrachain)
3271	*ν*(O–H) (weak hydrogen bond)
1784	*ν*(C=O) (anhydride)
1725	*ν*(C=O) (carboxyl)
1701	*ν*(C=O) (ester)
1656	*ν*(C=C) (unsaturated polycarboxylic acid)

**Table 2:** Gaussian calculation results of different molecules. AA: aconitic acid, HOMO: highest occupied molecular orbital, LUMO: lowest unoccupied molecular orbital.

Chemicals	HOMO (eV)	Location	LUMO (eV)	Location	ΔE (eV) ^1^
CA	−7.1830	O11 ^2^, O13	−0.2982	C1, C8, H18	6.0832
CA anhydride 1	−7.6040	O10, O12	−1.1045	C1, C4, C8	5.2769
CA anhydride 2	−7.8568	O5, O11	−1.1557	C1, C4, C7	5.2257
*cis*-AA	−7.5009	O11, O12	−3.3089	C1, C2, C4, C7	3.0725
*trans*-AA	−7.5311	O11, O12	−2.4831	C1, C2, C3, C4	3.8983
*trans*-AA anhydride 1	−8.0307	O5, O9, O11	−3.0393	C1, C2, C3, C4	3.3421
*trans*-AA anhydride 2	−8.0459	O6, O7	−3.0110	C1l, C2, C3, C5	3.3704
cellobiose	−6.3814	O14, O15, O17	1.1595	H36, H45	7.5409

^1^ The energy barrier between the LUMO of the molecule in this row and the HOMO of cellobiose. ^2^ The number of atoms can refer to the Scheme 1.
